# LZK-dependent stimulation of astrocyte reactivity promotes corticospinal axon sprouting

**DOI:** 10.3389/fncel.2022.969261

**Published:** 2022-09-15

**Authors:** Meifan Chen, Laura Ingle, Erik J. Plautz, Xiangmei Kong, Rui Tang, Neil Ghosh, Megan K. Romprey, William K. Fenske, Mark P. Goldberg

**Affiliations:** ^1^Spinal Cord and Brain Injury Research Center, College of Medicine, University of Kentucky, Lexington, KY, United States; ^2^Department of Neuroscience, College of Medicine, University of Kentucky, Lexington, KY, United States; ^3^Department of Neurology and Neurotherapeutics, University of Texas Southwestern Medical Center, Dallas, TX, United States; ^4^Department of Neurology, University of Texas Health Science Center San Antonio, San Antonio, TX, United States

**Keywords:** CNS injury, stroke, astrogliosis, reactive astrocytes, LZK/MAP3K13, axon plasticity, axon sprouting, CNTF

## Abstract

Injury to the adult mammalian central nervous system induces compensatory plasticity of spared axons—referred to as collateral axon sprouting—that can facilitate neural recovery. The contribution of reactive astrocytes to axon sprouting remains elusive. Here, we sought to investigate the role of axon degeneration-reactive astrocytes in the regulation of collateral axon sprouting that occurs in the mouse spinal cord after unilateral photothrombotic stroke of the primary motor cortex. We identified astrocytic leucine zipper-bearing kinase (LZK) as a positive regulator of astrocyte reactivity to corticospinal axon degeneration. Remarkably, genetic stimulation of astrocyte reactivity, via LZK overexpression in adult astrocytes, enhanced corticospinal axon sprouting. LZK promoted the production of astrocyte-derived ciliary neurotrophic factor (CNTF) that likely enhanced axon growth in mice with astrocytic LZK overexpression after injury. Our finding that LZK-dependent stimulation of astrocyte reactivity promotes corticospinal axon sprouting highlights the potential of engineering astrocytes to support injury-induced axon plasticity for neural repair.

## Introduction

Injury to the adult mammalian central nervous system (CNS) results in often permanent loss of motor and sensory functions, due to the lack of regenerative capacity of the damaged CNS. Limited spontaneous recovery, however, occurs in both experimental animal models and human survivors of CNS insults, including stroke and spinal cord injury (Hallett, 2001; Fawcett et al., 2007; Cramer, 2008; Courtine and Sofroniew, 2019). Such spontaneous neurological recovery is associated with injury-induced neural plasticity (Hallett, 2001; Cramer, 2008; Chen and Zheng, 2014; Hutson and Di Giovanni, 2019). One form of neural plasticity is the development of new collateral connections from intact axons, referred to as collateral axon sprouting, that occurs in the spinal cord (Lapash Daniels et al., 2009; Rosenzweig et al., 2010; Chen and Zheng, 2014; Starkey and Schwab, 2014; Carmichael et al., 2017). Intra-spinal sprouting of corticospinal tract (CST) axons, which are important for voluntary motor function, can form new synapses with denervated neuronal targets via direct or relay connections to regain motor control after injury (Bareyre et al., 2004; Rosenzweig et al., 2010; Ueno et al., 2012; Chen and Zheng, 2014; Lindau et al., 2014; Wahl et al., 2014). Experimentally stimulating CST axon sprouting, by increasing neuron-intrinsic potential of axon growth or blocking neuron-extrinsic inhibitors, improves functional recovery after CNS injury (Cafferty and Strittmatter, 2006; Starkey et al., 2012a; Ueno et al., 2012; Lindau et al., 2014; Wahl et al., 2014; Jin et al., 2015; Liu et al., 2017). However, the cellular and molecular events that coordinate axon sprouting remain elusive.

Astrocytes are important modifiers of CNS injury outcomes (Liddelow and Barres, 2017; Pekny et al., 2019). Following CNS injury, astrocytes undergo a range of molecular and functional changes that are collectively referred to as reactive astrogliosis (Sofroniew, 2014). Severe astrogliosis that results in a tissue-protective astrocytic scar at the site of acute focal injury has been extensively studied (Silver and Miller, 2004). Intriguingly, reactive astrocytes have also been suggested to play a role in intra-spinal CST axon sprouting that occurs distal to the injury site (Menet et al., 2003; Liu et al., 2014). Constitutive whole-body deletion of the cytoskeletal proteins glial fibrillary acidic protein and vimentin (GFAP^–/–^Vim^–/–^) attenuates astrocytes’ structural reactivity to injury, and reduces intra-spinal CST axon sprouting after unilateral cortical stroke but increases sprouting after spinal cord lateral hemisection (Menet et al., 2003; Liu et al., 2014). These studies raise the intriguing question of how reactive astrocytes regulate axon plasticity that occurs in undamaged tissue distal to the primary lesion. Given evidence from GFAP^–/–^Vim^–/–^ mice in the stroke model that suggests a positive role of reactive astrocytes in axon sprouting, would a gain-of-function approach that stimulates astrocyte reactivity promote neural plasticity and functional recovery?

Previously, we identified leucine zipper-bearing kinase (LZK) as an astrocyte-intrinsic, positive regulator of scar-forming astrogliosis at the injury site following spinal cord injury (Chen et al., 2018); we also identified LZK as an activator of STAT3 signaling that is critical to the regulation of astrogliosis (Okada et al., 2006; Herrmann et al., 2008; Chen et al., 2018). In this study, we tested the role of LZK in the regulation of axon degeneration-reactive astrocytes (Wang et al., 2009), which are non-scar forming. We hypothesized that these non-scar forming astrocytes serve as a cellular trigger of axon sprouting.

We used a photothrombotic ischemic model to unilaterally ablate the primary motor cortex containing CST neurons that control forelimb function. This lesion results in remote sprouting of uninjured contra-lesional CST axons in the cervical spinal cord that cross the midline into the denervated hemi-cord (Lapash Daniels et al., 2009; Lindau et al., 2014; Liu et al., 2014; Wahl et al., 2014; Kaiser et al., 2019). In this CST injury model, we deleted or overexpressed LZK in adult astrocytes to test the effects on i) astrocyte reactivity to CST degeneration in the spinal cord, ii) CST sprouting in the spinal cord, and iii) motor recovery. We found that astrocytic LZK is a positive regulator of astrocyte reactivity to CST axon degeneration in the spinal cord, where CST axon sprouting also occurs. While LZK gene deletion in adult astrocytes did not reduce injury-induced CST axon sprouting, stimulation of astrocyte reactivity via LZK overexpression in adult astrocytes enhanced CST axon sprouting. Furthermore, we found that LZK promotes the production of ciliary neurotrophic factor (CNTF), which is an activating ligand and transcriptional target of the JAK/STAT pathway sufficient to enhance CNS axon growth after injury. Our results reveal LZK-dependent, axon growth-supporting function of reactive astrocytes in undamaged tissue that may be harnessed to potentiate neural recovery.

## Results

### LZK is upregulated in astrocytes reactive to corticospinal axon degeneration

Our previous work identified LZK as a promoter of astrocytic scar formation at the injury site following spinal cord injury, and established genetic mouse models that enable tamoxifen-inducible LZK deletion (GFAP-CreER^T2^; LZK^f/f^) or LZK overexpression (GFAP-CreER^T2^; LZK^OE^) in adult astrocytes (Chen et al., 2018). Here, we sought to assess the contribution of axon degeneration-reactive astrocytes, which are non-scar forming, to CST axon sprouting. In order to do this, we first determined if LZK regulates axon degeneration-reactive astrocytes, which would then allow for their modulation via genetic manipulation of LZK expression (Chen et al., 2018).

**FIGURE 1:**
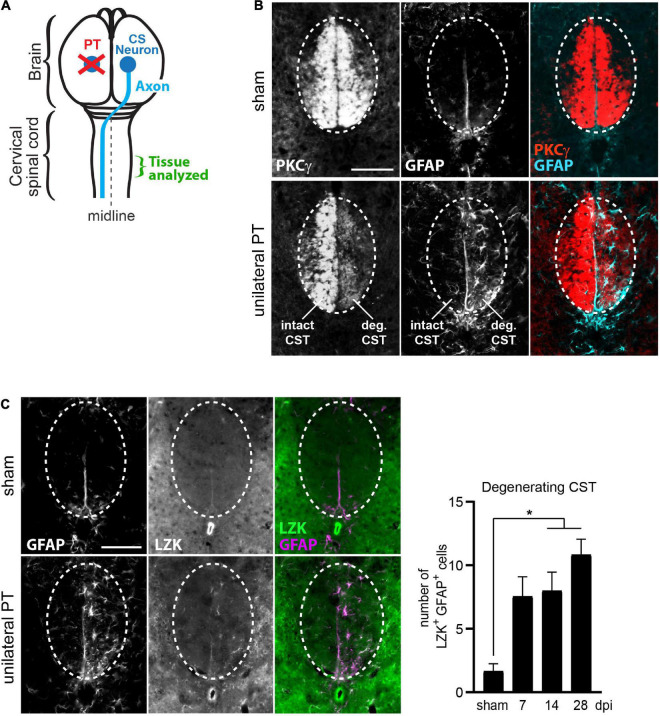
Upregulation of LZK expression in reactive astrocytes localized to the degenerating CST. **(A)** Illustration of unilateral photothrombosis (PT, red X) targeted to the forelimb sensorimotor cortex in one hemisphere of the mouse brain. Corticospinal (CS) axons (blue line) originating from CS neurons (blue circle) in the uninjured hemisphere decussate at the medulla and extend down the spinal cord, whereas CS axons originating from neurons ablated by photothrombosis undergo degeneration. Tissues from the cervical enlargement of the spinal cord at C7 were analyzed by immunohistochemistry in this study. **(B)** Immunofluorescence staining of PKCγ and GFAP in the corticospinal tract (CST) at the cervical spinal cords in wildtype mice without injury or 14 days post injury (dpi, unilateral PT). Dotted ovals outline the CST; intact and degenerating (deg) hemi-CSTs are indicated; scale bar represents 100 μm. **(C)** *Left*, immunofluorescence staining of endogenous LZK and GFAP in the CST at the cervical spinal cords of wildtype mice without injury or on 14 dpi. Dotted ovals outline the CST; scale bar represents 100 μm. *Right*, quantification of the number of LZK^+^GFAP^+^ cells localized to the degenerating CST in the cervical spinal cord on 7, 14, and 28 days after unilateral PT. *n* = 3 per timepoint; graphing mean with SEM. By one-way ANOVA, *p* < 0.05 for main injury effect. Asterisk (*) on graph indicates *p* < 0.05 for *post-hoc* comparison between sham and the post-injury timepoints of 14 dpi or 28 dpi with Bonferroni correction.

We utilized the unilateral photothrombotic (PT) stroke model to examine the interplay between axon degeneration-reactive astrocytes and CST axon sprouting (Figure 1A). Unilateral PT to the primary motor cortex is commonly used to experimentally study intra-spinal CST axon sprouting (Lapash Daniels et al., 2009; Lindau et al., 2014; Liu et al., 2014; Wahl et al., 2014; Kaiser et al., 2019). We targeted PT to the forelimb sensorimotor cortex unilaterally, which resulted in degeneration of CST axons originating from the ablated corticospinal neurons (CS neurons) and sprouting of intact CST axons originating from contra-lesional (uninjured) CS neurons (Figure 1A). CST axon degeneration and axon sprouting were examined at the cervical enlargement of the spinal cord, where forelimb-projecting CST axons innervate spinal motor neurons to control forelimb movement (Figure 1A).

In sham-injured mice, protein kinase C isoform gamma (PKCγ)—a marker of the CST—intensely labeled the intact CST main bundle in the dorsal funiculus of the cervical spinal cord (Figure 1B, *top* panel), with minimally detectable astrocytes positive for the reactivity marker GFAP (Figure 1B, *top* panel). Following unilateral PT stroke, degeneration of CST axons originating from the ablated neurons was evident by reduced PKCγ immunoreactivity on 14 days post injury (dpi), concurrent with the appearance of stellate GFAP^+^ reactive astrocytes (Figure 1B, *bottom* panel, “deg CST”). Furthermore, these axon degeneration-reactive astrocytes upregulated endogenous LZK protein expression (Figure 1C, *bottom* panel). Compared to the intact CST, the number of LZK^+^GFAP^+^ cells in the degenerating CST increased by four-fold on 7 dpi, and sustained until at least 28 dpi (Figure 1C, graph). These findings suggest a positive role of LZK in astrocyte reactivity to CST axon degeneration, and that manipulation of LZK gene expression may modulate such reactivity.

### LZK is a positive regulator of astrocyte reactivity to corticospinal tract axon degeneration

To test LZK-dependent regulation of astrocyte reactivity to axon degeneration, we used GFAP-CreER^T2^; LZK^f/f^ mice (herein referred to as astrocytic LZK-KO mice) and GFAP-CreER^T2^; LZK^OE^ mice (herein referred to as astrocytic LZK-OE mice) that allow tamoxifen-inducible LZK deletion or overexpression in adult astrocytes, respectively (Chen et al., 2018). Tamoxifen treatment was given one week prior to unilateral PT. Tamoxifen-treated GFAP-CreER^T2^ littermates were used as genotype controls.

**FIGURE 2:**
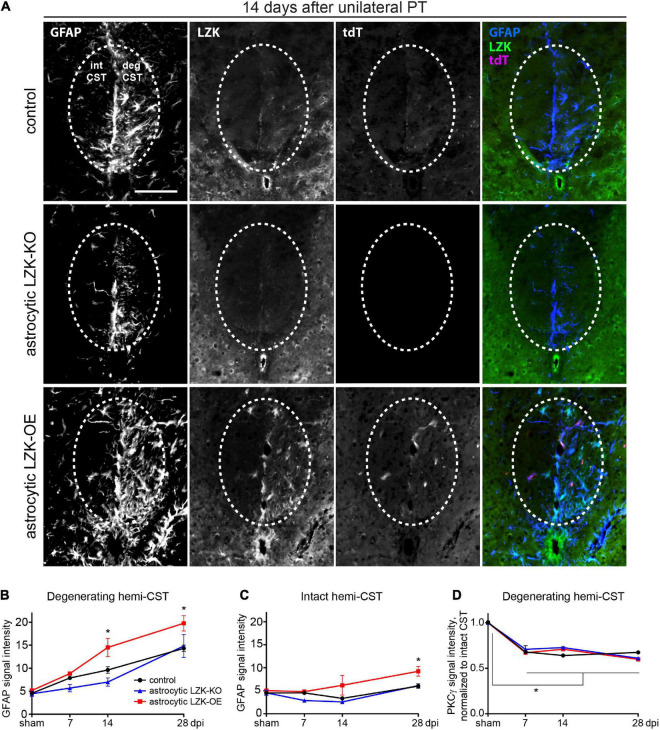
LZK is a positive regulator of astrocyte reactivity to CST axon degeneration. **(A)** Immunofluorescence staining of LZK and GFAP in the CST at the cervical spinal cords of tamoxifen treated genotype control, astrocytic LZK-KO (GFAP-CreER^T2^; LZK^f/f^), and astrocytic LZK-OE (GFAP-CreER^T2^; LZK^OE^) mice; 14 days after unilateral PT. Dotted ovals outline the CST; intact and degenerating (deg) hemi-CSTs are indicated in the upper left panel that applies to all images; scale bar represents 100 μm. **(B)** Quantification of astrocyte reactivity within the degenerating hemi-CST, based on GFAP immunofluorescence intensity, in tamoxifen treated genotype control, astrocytic LZK-KO (GFAP-CreER^T2^; LZK^f/f^), and astrocytic LZK-OE (GFAP-CreER^T2^; LZK^OE^) mice without injury and on 7, 14, and 28 days after unilateral PT. *n* = 3 per genotype per timepoint; graphing group mean with SEM. By two-way ANOVA, *p* < 0.05 for main injury effect and genotype effect, no interaction. Asterisk (*) on graph indicates *p* < 0.05 for *post-hoc* comparison between genotype control and GFAP-CreER^T2^; LZK^OE^ mice at the corresponding timepoints with Bonferroni correction. Not marked on graph: *p* < 0.05 for the following *post-hoc* comparisons of injury effect within each genotype with Bonferroni correction (control—sham vs 14 dpi or 28 dpi; astrocytic LZK-KO—sham vs 28 dpi; astrocytic LZK-OE—sham vs 14 dpi or 28 dpi). **(C)** Quantification of astrocyte reactivity within the intact hemi-CST, based on GFAP immunofluorescence intensity, in the indicated tamoxifen treated groups over a time course after unilateral PT. *n* = 3 per genotype per timepoint; graphing group mean with SEM. By two-way ANOVA, *p* < 0.05 for main injury effect and genotype effect, no interaction. Asterisk (*) on graph indicates *p* < 0.05 for *post-hoc* comparison between genotype control and GFAP-CreER^T2^; LZK^OE^ mice at the corresponding timepoint with Bonferroni correction. Not marked on graph: *p* < 0.05 between sham vs 28 dpi within LZK-OE group, by *post-hoc* comparison with Bonferroni correction. **(D)** Quantification of CST axon degeneration within the degenerating hemi-CST, based on PKCγ immunofluorescence intensity, in the indicated tamoxifen treated groups over a time course after unilateral PT. *n* = 3 per genotype per timepoint; graphing group mean with SEM. By two-way ANOVA, *p* < 0.05 for injury effect only. Asterisk (*) on graph indicates *p* < 0.05 for *post-hoc* comparison in PKCγ signal intensity between sham injury and all post-injury timepoints with Bonferroni correction.

We compared astrocyte reactivity to CST axon degeneration based on GFAP immunoreactivity among control, astrocytic LZK-KO, and astrocytic LZK-OE mice in a time course after unilateral PT. In genotype control mice, astrocyte reactivity localized to the degenerating CST increased by 1.5-fold on 7 dpi that further increased by 2–3 fold on 14–28 dpi respectively, compared to sham baseline (Figures 2A,B). These axon degeneration-reactive astrocytes also expressed LZK (Figures 1C, 2A). Astrocytic LZK deletion did not affect the baseline level of GFAP immunoreactivity in the CST without injury (Figure 2B). Following unilateral PT, astrocytic LZK-KO mice showed reduced LZK expression and reactive astrocyte density within the degenerating CST (Figure 2A), though exhibited only a statistically non-significant trend toward lower astrocyte reactivity to CST axon degeneration compared to the genotype control on 7 and 14 dpi (Figure 2B).

In contrast, astrocytic LZK-OE mice showed 50% increase in astrocyte reactivity to CST axon degeneration that was sustained from 14 to 28 dpi, compared to the genotype control at the same timepoints (Figures 2A,B). This was accompanied by a strong upregulation of LZK and its fluorescent reporter tdTomato in GFAP^+^ cells (Chen et al., 2018; Figure 2A). Interestingly, in spite of this robust stimulation of astrocyte reactivity to CST axon degeneration, there was no difference in baseline astrocyte reactivity within the CST between control and astrocytic LZK-OE mice in the absence of injury (Figures 2B,C). This suggests astrocytic LZK-OE alone is insufficient to induce astrocyte reactivity in the healthy CST without injury.

Following unilateral PT, astrocyte reactivity in the spinal cord was largely localized to the degenerating CST. The level of GFAP immunoreactivity in the spared hemi-CST did not change from sham baseline in the control and astrocytic LZK-KO mice throughout the time course (Figure 2C). Minimal increase was observed in astrocytic LZK-OE mice on 28 dpi (Figure 2C). Lastly, the level of CST degeneration in the cervical spinal cord was verified to be comparable across all genotypes, based on quantification of PKCγ immunoreactivity in the degenerating hemi-CST in the cervical spinal cord (Figure 2D), indicating consistency in injury severity in all animals. Taken together, these findings demonstrate that following unilateral PT stroke to the primary motor cortex, while astrocytic LZK deletion did not abrogate reactive astrocytes localized to the degenerating CST, astrocytic LZK overexpression amplified astrocyte reactivity to CST axon degeneration in the spinal cord.

### Astrocytic LZK overexpression enhances intra-spinal corticospinal tract axonal sprouting

**FIGURE 3:**
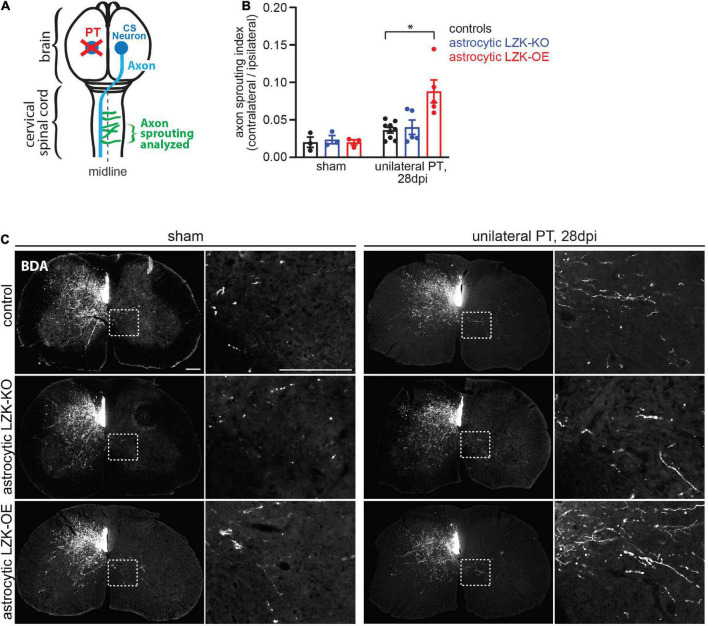
LZK overexpression in astrocytes enhanced CST axon sprouting. **(A)** Diagram shows sprouting of intact CST axons (green) that cross the midline (dotted line) in the spinal cord, following unilateral PT (red X) to the forelimb sensorimotor cortex. Tissues from the cervical spinal cord at C7 were used to analyze CST axon sprouting. **(B)** Quantification of CST axon sprouting density index (contra-lesional/ipsi-lesional) in the indicated tamoxifen treated groups. *n* = 3 per group without injury; *n* = 5–9 per group 28 days after unilateral PT; graphing group mean with SEM. By two-way ANOVA, *p* < 0.05 for main injury effect and genotype effect, *p* < 0.05 for interaction between the two variables. Asterisk (*) on graph indicates *p* < 0.05 for *post-hoc* comparison between genotype control and GFAP-CreER^T2^; LZK^OE^ mice on 28 dpi with Bonferroni correction. **(C)** Biotinylated dextran amine (BDA) was injected into the uninjured cerebral cortex to label intact CST axons in the cervical spinal cord in the indicated tamoxifen treated groups, without or after unilateral PT. Dotted box outlines area magnified in adjacent image to show CST axon sprouting across the midline into the denervated gray matter, quantified in panel **(B)**. Scale bar represents 200 μm in both low and high magnification images.

Given that LZK promotes astrocyte reactivity to CST axon degeneration in the spinal cord, we next used astrocytic LZK mutant mice to determine the effects of modulating axon degeneration-reactive astrocytes on intra-spinal CST axon sprouting following unilateral PT. As before, LZK gene manipulation in adult astrocytes was achieved by tamoxifen treatment one week prior to unilateral PT. Two weeks after injury, the neuronal tracer biotinylated dextran amine (BDA) was injected into the contra-lesional forelimb sensorimotor cortex to label CST neurons that project to the cervical spinal cord. At the end of a 4 weeks survival period after PT, CST axon sprouting across the midline in the cervical enlargement of the spinal cord was analyzed by BDA immunostaining (Figure 3A).

In the absence of injury, baseline intra-spinal CST axon sprouting was similar among all genotypes (Figures 3B,C). Unilateral PT induced CST axon sprouting by approximately two-fold in genotype control and likewise in astrocytic LZK-KO mice on 28 dpi, compared to their respective no injury baseline (Figures 3B,C). The lack of effect of astrocytic LZK deletion on CST axon sprouting correlates with its inability to abrogate astrocyte reactivity to CST axon degeneration (Figures 2A,B, 3B,C). In contrast, astrocytic LZK-OE mice exhibited a four-fold increase in injury-induced CST axon sprouting compared to its own sham baseline (Figures 3B,C), which is a two-fold enhancement compared to the injured genotype control or astrocytic LZK-KO mice (Figures 3B,C). Notably, the ability of astrocytic LZK overexpression to augment CST axon sprouting requires the presence of injury (Figures 3B,C). The stimulatory effects of astrocytic LZK overexpression on CST axon sprouting positively corresponds to its ability to amplify astrocyte reactivity to CST axon degeneration after unilateral PT (Figures 2A,B, 3B,C). Taken together, these results show that LZK deletion in adult astrocytes does not allow for testing of the role of axon degeneration-reactive astrocytes in axon sprouting by loss-of-function approach; whereas amplification of astrocyte reactivity by LZK overexpression in adult astrocytes stimulates axon sprouting but only in the presence of injury.

### Astrocytic LZK overexpression trends toward improved motor coordination, but not skilled stepping

**FIGURE 4:**
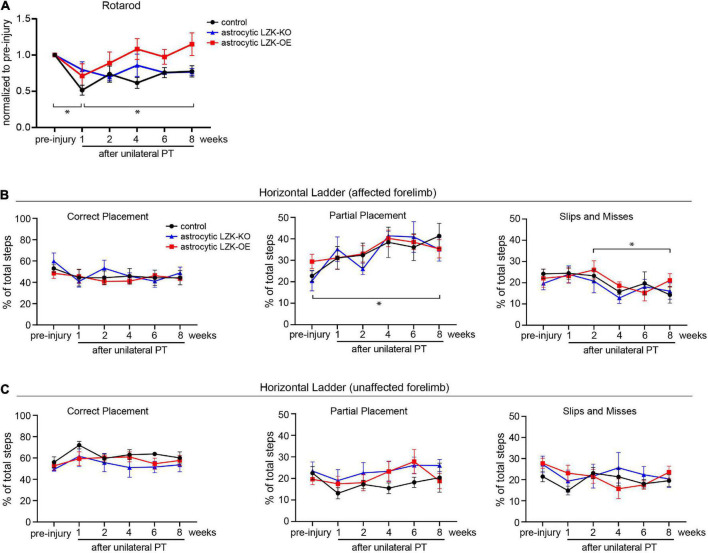
LZK overexpression in astrocytes trended toward improved recovery of general motor coordination, but not skilled stepping. **(A)** General motor coordination and balance were assessed by rotarod in the indicated tamoxifen treated groups before and over 8 weeks after unilateral PT. Time on rotarod of each animal was normalized to its own pre-injury performance. *n* = 6 per group; graphing group mean with SEM. By two-way ANOVA with repeated measures, *p* < 0.05 for injury effect and *p* = 0.0501 for genotype effect. Asterisks (*) on graph indicate *p* < 0.05 for *post-hoc* comparison of rotarod performance at the indicated time points in the control group with Bonferroni correction. **(B)** Skilled stepping of the affected forelimb, contralateral to PT, assessed by regular horizontal ladder before and over 8 weeks after unilateral PT. *n* = 6 per group; graphing group mean with SEM. *Left*, percentage of total steps that correctly placed on ladder rungs. *Middle*, percentage of total steps that partially placed on ladder rungs; two-way ANOVA with repeated measures showed *p* < 0.05 for injury effect only; asterisk (*) on graph indicates *p* < 0.05 for *post-hoc* comparison between pre-injury and week 8 after unilateral PT in the control group with Bonferroni correction. *Right*, percentage of total steps that were slips or misses; two-way ANOVA with repeated measures showed *p* < 0.05 for injury effect only; asterisk (*) on graph indicates *p* < 0.05 for *post-hoc* comparison between weeks 2 and 8 after unilateral PT in the control group with Bonferroni correction. **(C)** Skilled stepping of the unaffected forelimb, ipsilateral to PT, assessed by regular horizontal ladder as in panel **(B)**.

Because CST axon sprouting can facilitate functional recovery, we next assessed motor recovery in control, astrocytic LZK-KO, and astrocytic LZK-OE mice following unilateral PT. We first utilized the rotarod test to examine general motor coordination, balance, and endurance over 8 weeks of recovery period. Unilateral PT resulted in 30–50% deficit in rotarod performance at 1 week after injury (Figure 4A). Compared to genotype control and astrocytic LZK-KO mice that reached 76–78% of their pre-injury performance by 8 weeks after injury respectively, astrocytic LZK-OE mice showed a statistically non-significant trend toward full recovery to their pre-injury level by 8 weeks (Figure 4A).

In addition to assessing gross motor function, we also examined skilled forelimb stepping by regular horizontal ladder. The ladder task detects limb-specific, subtle or chronic deficits in paw placement accuracy and compensatory adjustments (Farr et al., 2006). No difference at baseline performance was observed among control, astrocytic LZK-KO, and astrocytic LZK-OE mice by ladder assessment (Figure 4B). Unilateral PT resulted in subtle deficit and adaptive use of the affected forelimb, shown by a two-fold increase of partially placed steps taken by the affected forelimb at 8 weeks after injury compared to pre-injury baseline in the control group (Figure 4B, *middle* graph). Correspondingly, there was a decrease in correctly placed steps (did not reach statistical significance, Figure 4B, *left* graph) and also a decrease in slips/misses (statistically significant, Figure 4B, *right* graph) following unilateral PT. This injury-dependent adaptation to partial placement in stepping of the affected forelimb was similar across genotypes. No statistically significant change in stepping behavior was observed in the unaffected forelimb (Figure 4C). Taken together, astrocytic LZK-OE mice showed a statistically non-significant trend toward improved recovery of general motor coordination as assessed by rotarod, but did not affect skilled forelimb stepping on horizontal ladder.

### LZK promotes production of ciliary neurotrophic factor by astrocytes

We next sought to determine the molecular basis of LZK’s ability to augment CST axon sprouting. Our previous work identified LZK as an activator of signal transducer and activator of transcription 3 (STAT3) (Chen et al., 2018). One of the transcriptional targets of STAT3 is the secreted neurotrophic cytokine ciliary neurotrophic factor (CNTF) (Keasey et al., 2013), which is in turn an activating ligand of the JAK-STAT pathway (Stahl et al., 1994). Additionally, intra-spinal AAV-mediated overexpression of CNTF is sufficient to increase sprouting of CST axons in the cervical spinal cord following unilateral transection of the CST at the brainstem (Jin et al., 2015).

**FIGURE 5:**
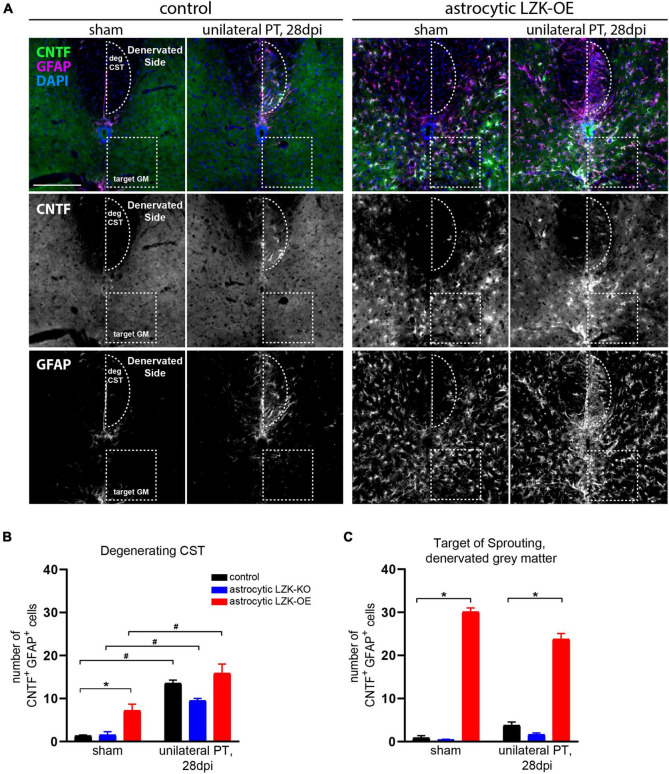
Upregulation of astrocyte-derived CNTF following astrocytic LZK overexpression. **(A)** Immunofluorescence staining of CNTF and GFAP in the indicated tamoxifen treated groups, with no injury or 28 days after unilateral PT. Dotted semi-oval outlines the degenerating hemi-CST (labeled as “deg CST”) at the cervical spinal cord; dotted box outlines the gray matter in the denervated spinal cord that intact CST axons sprout into (labeled as “target GM,” on the “Denervated Side”). Scale bar represents 200 μm. **(B)** Quantification of the number of CNTF^+^GFAP^+^ cells in the degenerating CST [outlined by dotted semi-oval in panel **(A)**], in the groups indicated, with no injury or 28 days after unilateral PT. *n* = 3 per group; graphing group mean with SEM. By two-way ANOVA, *p* < 0.05 for main injury effect and genotype effect, no interaction. In the sham injury group, asterisk (*) indicates *p* < 0.05 for *post-hoc* comparison between genotype control and GFAP-CreER^T2^; LZK^OE^ mice with Bonferroni correction. Pound sign (#) indicates *p* < 0.05 between sham and 28 dpi within each genotype by *post-hoc* comparisons with Bonferroni correction. Not marked on graph: *p* < 0.05 between astrocytic LZK-KO and LZK-OE mice without injury, by *post-hoc* comparison Bonferroni correction. **(C)** Quantification of the number of CNTF^+^GFAP^+^ cells in the sprouting target gray matter [outlined by dotted box in panel **(A)**], in the groups indicated, with no injury or 28 days after unilateral PT. *n* = 3 per group; graphing group mean with SEM. By two-way ANOVA, *p* < 0.05 for main genotype effect only. Asterisk (*) indicates *p* < 0.05 for *post-hoc* comparison between genotype control and GFAP-CreER^T2^; LZK^OE^ mice at the corresponding timepoint with Bonferroni correction. Not marked on graph: *p* < 0.05 between astrocytic LZK-KO and LZK-OE mice without injury or on 28 dpi, by *post-hoc* comparison Bonferroni correction.

Based on this evidence supporting LZK as an upstream regulator of CNTF, we tested if astrocytes in LZK-OE mice produce CNTF following unilateral PT. We first examined CNTF expression in astrocytes reactive to CST axon degeneration in the cervical cord. In genotype controls, unilateral PT induced a ten-fold increase in the number of CNTF^+^GFAP^+^ cells within the degenerating CST (Figures 5A,B). Astrocytic LZK deletion reached only a six-fold increase by the same measurement, but this reduction compared to the genotype control did not reach statistical significance (Figure 5B). While astrocytic LZK overexpression increased the number of CNTF^+^GFAP^+^ cells within the CST at no injury baseline, the number of CNTF^+^GFAP^+^ cells localized to the degenerating CST was comparable to that observed in genotype control (Figures 5A,B). These results suggest that first, astrocytes reactive to axon degeneration express CNTF and therefore have the potential to support axon growth; second, CNTF-producing reactive astrocytes within the degenerating CST, which were present in similar numbers in genotype control and LZK-OE mice, likely do not account for the ability of astrocytic LZK overexpression to enhance CST axon sprouting.

Next, we examined CNTF expression in astrocytes in the spinal gray matter on the denervated side of the spinal cord, where sprouting CST axons cross the midline just below the dorsal column (Figures 3C, 5A). Within this sprouting target in the gray matter, the genotype control had minimally detectable number of CNTF^+^GFAP^+^ cells in the absence or presence of unilateral PT, likewise for the astrocytic LZK-KO group (Figures 5A,C). In contrast, astrocytic LZK overexpression significantly increased the number of CNTF^+^GFAP^+^ cells within the sprouting target gray matter, by more than six-fold compared to the genotype control at either sham baseline, or following unilateral PT (Figures 5A,C). Notably, such increase of CNTF-producing astrocytes was not limited to the sprouting target gray matter, but observed throughout the spinal gray matter of astrocytic LZK-OE mice (Figures 5A,C). The unique presence of CNTF-producing astrocytes exclusively in the spinal gray matter of astrocytic LZK-OE mice presents a potential mechanism that promotes axon sprouting in these animals. Despite widespread CNTF-producing astrocytes in the gray matter of astrocytic LZK-OE mice, CST axon sprouting only occurred in the presence of injury (Figures 3B,C). These findings together demonstrate that LZK overexpression in adult astrocytes stimulates non-scar forming reactive astrocytes that produce CNTF in the spinal gray matter, which may contribute to enhance CST axon sprouting in astrocytic LZK-OE mice in the presence of unilateral PT stroke.

## Discussion

Understanding how astrocytes impact axon growth following injury will open new avenues to potentiate axon plasticity and neural recovery. Current knowledge of reactive astrocytes in acute focal injury is largely based on studies that focused on scar-forming astrocytes at the injury site, which are part of a multicellular glial scar traditionally recognized as inhibitory to axon regeneration in the adult mammalian CNS (Davies et al., 1999; Silver and Miller, 2004; Sofroniew, 2014; Hara et al., 2017; Stern et al., 2021), though regeneration-supporting astrocytes have also been observed at the primary lesion (Lee et al., 2010; Liu et al., 2010; Zukor et al., 2013; Du et al., 2015; Anderson et al., 2016).

Two studies have implicated a role of astrocytes in axon sprouting that occurs distal to the injury site, using mice with constitutive and whole-body knockout of two cytoskeletal proteins GFAP and vimentin (GFAP^–/–^Vim^–/–^) (Menet et al., 2003; Liu et al., 2014). However, it remained elusive the extent to which astrocyte reactivity *per se* impacts axon sprouting, where or how astrocytes regulate axon sprouting in undamaged tissue, and what effects amplification of astrocyte reactivity would have on axon sprouting.

In this study, we sought to test the role of axon degeneration-reactive astrocytes in the regulation of compensatory axon sprouting that occurs in undamaged tissue away from the injury site. We targeted PT stroke to the primary motor cortex unilaterally to induce CST axon degeneration and collateral CST axon sprouting in the spinal cord. We first determined LZK-dependent regulation of astrocyte reactivity to axon degeneration, using GFAP-CreERT2; LZK^f/f^ (astrocytic LZK-KO) mice and GFAP-CreERT2; LZK^OE^ (astrocytic LZK-OE) mice to respectively delete or overexpress LZK—a key signaling activator of the astrocytic injury response—in adult astrocytes (Chen et al., 2018). Astrocytic LZK deletion did not abolish reactive astrocytes localized to the degenerating CST, and therefore did not allow for testing of the requirement of these reactive astrocytes in CST axon sprouting. In contrast, astrocytic LZK overexpression enhanced astrocyte reactivity to CST axon degeneration and collateral CST axon sprouting. However, because astrocytic LZK overexpression also induced stellate reactive astrocytes throughout the spinal gray matter (Chen et al., 2018) in addition to the degenerating CST, reactive astrocytes from both of these regions may contribute to stimulating axon sprouting in astrocytic LZK-OE mice. Nevertheless, these findings provide exciting first insights into the positive effects of LZK-induced reactive astrocytes on axon plasticity in an injured CNS.

Our previous discovery of LZK as an activator of STAT3 signaling in astrocytes (Chen et al., 2018) guided our identification of astrocyte-derived CNTF as a candidate molecular effector of LZK that promotes axon sprouting. In CNS neurons, STAT3 activation potently stimulates axon regeneration and sprouting after CNS injury (Sun et al., 2011; Lang et al., 2013; Jin et al., 2015), which is in part mediated by the neurotrophic cytokine CNTF (Jin et al., 2015). CNTF alone is sufficient to promote intra-spinal CST axon sprouting (Jin et al., 2015). We found that astrocytes reactive to CST axon degeneration upregulate LZK and CNTF, suggesting an endogenous capacity of these astrocytes to support axon growth. These CNTF-producing astrocytes reactive to axon degeneration may serve as an endogenous cellular mechanism that promotes spontaneous intra-spinal CST axon sprouting following CNS injury. Production of CNTF by reactive astrocytes *in vivo* also has been observed in other injury models (Park et al., 2000; Muller et al., 2007; Tripathi and McTigue, 2008), and reported to stimulate axon regeneration (Muller et al., 2007).

Within the degenerating CST, astrocytic LZK overexpression did not further increase CNTF-producing astrocytes compared to genotype control. This suggests that CNTF produced by astrocytes in the degenerating CST may not account for LZK-dependent stimulatory effects on axon sprouting. Rather, it may be the unique presence of CNTF-producing astrocytes in the spinal gray matter of astrocytic LZK-OE mice that potentiated CST axon sprouting. This environment rich in astrocyte-derived CNTF resulting from astrocytic LZK overexpression is analogous to intra-spinal AAV-mediated CNTF overexpression reported to augment CST axon sprouting (Jin et al., 2015). Strikingly, astrocytic LZK-OE alone was insufficient to induce CST axon sprouting in the absence of injury. This indicates a requirement for injury-dependent events, such as the loss of neuronal activity (Jiang et al., 2019), to elicit axon growth-enhancing effects of astrocytic LZK overexpression.

In addition to sprouting of CST axons originating from the contra-lesional motor cortex, which we examined here, there are other forms of axon plasticity occurring in the spinal cord that are associated with motor recovery following unilateral cortical injury. For example, CST axons originating from the ipsi-lesional hindlimb motor cortex sprout into the cervical spinal cord following unilateral ischemic injury targeted to the forelimb motor cortex (Starkey et al., 2012b). Several descending brainstem-spinal tracts have also been observed to sprout into the denervated spinal cord. It was estimated that compared to CST neurons, six times more brainstem-spinal neurons project into the affected cervical hemicord following unilateral PT (Bachmann et al., 2014). Brainstem neurons of the medullary raphe and reticular nuclei have been proposed to form relay pathways to enable control of the denervated neuronal targets in the spinal cord by the spared motor cortex (Bachmann et al., 2014). It is possible that these descending tracts can also respond to LZK-induced astrocyte reactivity.

While astrocytic LZK overexpression increased CST axon sprouting, it did not result in statistically significant improvement in post-stroke motor recovery as assessed by rotarod and horizontal ladder. Although postural coordination and skilled paw placement measured by these two tests are commonly used to evaluate motor recovery after unilateral stroke, tests that are more sensitive such as detailed kinematic analysis of forelimb movement, or tests that are more specific to CST-mediated fine motor control such as single pellet grasping may be needed to detect functional alterations in mice with astrocytic LZK overexpression. Additionally, while enhancing axon sprouting is important to promoting neural plasticity after injury, itself alone may not be adequate to result in meaningful functional restoration. Timing of rehabilitative training relative to administration of axon growth-enhancing intervention has been shown to critically affect the motor recovery after cortical stroke modeled by unilateral PT (Wahl et al., 2014). Incorporation of timely rehabilitative training therefore may be required to effect motor improvement in astrocytic LZK-OE mice.

Together with our previous work, we have determined LZK as a positive regulator of two distinct forms of astrocyte reactivity: (i) scar-forming reactive astrocytes at the site of acute focal trauma (Chen et al., 2018); and (ii) non-scar forming reactive astrocytes that are either endogenously responding to axon degeneration or exogenously induced by astrocytic LZK overexpression in undamaged tissue. Our findings point to the capacity of the latter form of reactive astrocytes in supporting axon sprouting. The injury response and physiological impacts of astrocytes are context-dependent, influenced by CNS region, the type of insult, stage of injury, and the local injury environment among other variables (Zhang and Barres, 2010; Zamanian et al., 2012; Khakh and Sofroniew, 2015; Diaz-Castro et al., 2021; Hasel et al., 2021; Burda et al., 2022). While LZK induces astrocyte reactivity as broadly assessed by traditional hallmarks of reactivity here and previously (Chen et al., 2018), it does not mean that reactive astrocytes in general promote axon growth. It remains to be elucidated whether LZK-stimulated astrocytes represent a specific subtype of reactive astrocytes. Their axon growth-supportive potential is likely contextual, given the multitude of factors mentioned above that influence astrocyte functions. The level of LZK induction in astrocytes—whether occurring endogenously following injury or by experimental manipulation—is also an important consideration, as a strong genetic induction of LZK overexpression results in poor survival (Chen et al., 2018). This indicates a delicate balance in eliciting LZK-dependent astrocyte reactivity that is pro-regenerative versus dysfunctional.

The ability of LZK overexpression in adult astrocytes to upregulate CNTF production and stimulate CST axon sprouting highlight the potential of engineering astrocytes for neural repair. CNTF has also been shown to promote survival and proliferation of oligodendrocyte precursor cells (Barres et al., 1996; Linker et al., 2002) and protect the integrity of myelin sheaths in mouse model of multiple sclerosis (Linker et al., 2002). Enhancing axon plasticity in the presence of CNTF, together with interventions that strengthen appropriate reinnervation, would be beneficial to CNS repair not only following acute focal trauma, but conceivably also in CNS diseases involving axonopathy and inflammatory demyelination.

### Experimental procedures

**Mice**. All mouse husbandry and experimental procedures were performed in compliance with protocols approved by the Institutional Animal Care and Use Committee at the University of Texas Southwestern Medical and the University of Kentucky. Tamoxifen inducible and astrocyte-targeted LZK-knockout mice (GFAP-CreER*^T2^*;LZK^f/f^) and LZK-overexpression mice (GFAP-CreER*^T2^*;LZK^OE^) have been previously described (Chen et al., 2018). Both lines were bred into C57BL/6 background (*N* > 10 generations) for this study. Adult male and female mice at the age of 10–12 weeks were used. To delete LZK from astrocytes in adult GFAP-CreER*^T2^*;LZK^f/f^ mice, 75 mg/kg of tamoxifen was given by oral gavage once daily, consecutively for five days. To induce LZK overexpression in astrocytes of adult GFAP-CreER*^T2^*;LZK^OE^ mice, 75 mg/kg of tamoxifen was given by oral gavage once daily, consecutively for two days. Tamoxifen dosage was reduced in GFAP-CreER*^T2^*;LZK^OE^ to prevent severe astrogliosis-associated lethality (Chen et al., 2018). One week after the last dose of tamoxifen treatment, all mice were subjected to unilateral cortical photothrombosis. All surgical procedures, animal dissection, tissue processing, and data analyses were performed blind to genotypes.

**Photothrombotic stroke**. Unilateral photothrombotic stroke was performed similarly as previously described (Watson et al., 1985; Poinsatte et al., 2019; Becker et al., 2020). Mice were anesthetized with isoflurane (3.5% induction, 2% maintenance in 70% nitrous oxide and 30% oxygen mixture). While fixed in a stereotactic frame (Kopf), the mouse scalp was injected with lidocaine and incised at the midline to expose the intact skull. Rose Bengal (40 mg/kg, 5 mg/ml in saline, Sigma) was injected intraperitoneally one minute before delivery of focused 561 nm laser illumination (Coherent, laser diameter of 3 mm) to the forelimb motor cortex centered at 1.7 mm lateral to bregma under a surgical microscope (Nikon SMZ800), at 45 mW for 15 min. Sham-injured mice received anesthesia, analgesia, scalp incision, Rose Bengal injection, but no laser illumination.

**Anterograde labeling of CST axons**. Two weeks after photothrombosis, mice used for analyses of CST axon sprouting received BDA injections to anterogradely trace forelimb targeting CST axons originating from the contra-lesional sensorimotor cortex. 0.5 μl of 10% BDA in saline (10,000 MW, Invitrogen D1956) was injected into each of three sites: (AP, ML) in mm from Bregma: (0.5, 1.25), (−0.5, 1.25), (0, 2) at the depth of 0.6 mm. Two weeks after BDA injection (equivalent to 4 weeks after photothrombosis), mice were euthanized for brain and spinal cord dissections.

**Histology.** Terminal anesthesia was performed by isoflurane overdose. Mice were perfused with 4% paraformaldehyde intracardially, after which brains and spinal cords were dissected and post-fixed at 4°C overnight. Tissues were cryoprotected in 30% sucrose at 4°C overnight before embedding. C4-C7 spinal cord was embedded for transverse sections in O.C.T compound (Scigen Tissue-Plus) on dry ice. Embedded tissue blocks were sectioned at 15 μm thickness on a cryostat (Leica). Free floating sections were collected in PBS with 0.01% sodium azide for further histological examination.

**Immunohistochemistry.** Staining procedures have been described previously (Chen et al., 2018). Free floating sections were washed twice (0.2% triton in PBS, for 10 min), then blocked and permeabilized (0.4% triton, 5% sera matching species of secondary antibody in PBS) for 1 h at room temperature. Primary antibody incubation (see antibody concentrations below, in 0.2% triton, 1% matching sera, 0.01% sodium azide, in PBS) was carried out at room temperature overnight on a rocking platform. Sections were washed three times and incubated in secondary antibody solution (all secondary antibodies used at 1:500) for 2 h at room temperature on a rocking platform. After three washes, sections were incubated with DAPI (1 μg/ml in PBS) for 10 min, then mounted onto glass slides (Fisher Scientific) and cover slipped with Fluoromount-G (Southern Biotech). BDA staining was done as previously described (Geoffroy et al., 2015). Coronal sections of the cervical spinal cord were incubated in Vectastain ABC solution (Vector Laboratories) at 4°C overnight, washed three times in PBS, then incubated with TSA Plus solution at 1:200 (Perkin Elmer, see below) according to manufacturer’s instructions for 10 min, protected from light at room temperature.

**Antibodies.** Commercially available primary antibodies used were: LZK (1:500, rabbit, Sigma-Aldrich HPA016497), GFAP (1:1,000, rabbit, Dako Z0334), GFAP (1:1,000, rat, Invitrogen 2.2B10), CNTF (1:300, goat, Novus AF-557-SP), PKCγ (1:1,000, mouse, Santa Cruz SC166385). Alexa Fluor-tagged secondary antibodies used were Alexa 488, Alexa 546, and Alexa 647. BDA detection was performed using TSA Plus Cyanine 5 or Cyanine 3 (Perkin Elmer).

**Microscopy and quantification.** Stained tissue sections were imaged by an epifluorescent slide-scanning microscope (Zeiss Axio Scan.Z1). Image analyses were done using ImageJ software. All quantifications were conducted by observers blind to genotypes. Images of transverse sections from the cervical spinal cord at levels C7 were used for the following quantifications. For exact values of n per group, see figure legends.

To quantify PKCγ immunofluorescence intensity of the degenerating hemi-CST (area labeled as “deg CST” in Figure 1B) in the cervical spinal cord, integrated density within a semi-oval region of interest covering the degenerating hemi-CST (about 25,000 μm^2^) was normalized to that of the intact hemi-CST (area labeled as “deg CST” in Figure 1B). Four transverse sections of C7 spinal cord per animal were averaged to generate PKCγ signal intensity per animal. To quantify GFAP immunofluorescence intensity of the degenerating hemi-CST (area labeled as “deg CST” in Figure 1B) or intact hemi-CST (area labeled as “intact CST” in Figure 1B), integrated density within a semi-oval region of interest covering the respective hemi-CST (about 25,000 μm^2^) was averaged over five transverse cervical spinal cord sections per animal after subtraction of background signal.

For LZK^+^GFAP^+^ cell count in the degenerating hemi-CST, the number of cells double positive for LZK and GFAP by co-immunofluorescence staining was quantified within the semi-oval region labeled as “deg CST” (Figures 1B,C), where reactive astrocytes localized. Three transverse sections of C7 spinal cord per animal were averaged to generate cell count per animal. For CNTF^+^GFAP^+^ cell count in the degenerating hemi-CST, the number of cells double positive for CNTF and GFAP by co-immunofluorescence staining was quantified within the region outlined as “deg CST” in Figure 5A (area of 25,837 μm^2^). Three transverse cervical spinal cord sections per animal were averaged to generate cell count per animal. Likewise, CNTF^+^GFAP^+^ cells in the sprouting target area of the denervated gray matter were quantified within the region outlined as “target GM” in Figure 5A (area of 40,000 μm^2^).

CST axon sprouting was quantified as previously described (Grider et al., 2006). Density of BDA-labeled axons on the denervated side of the spinal cord was quantified within a rectangular area of 0.54 mm^2^ in transverse sections of C7 spinal cord, and normalized against the axon density on the innervated side of the spinal cord of the same sections. Axons in the main CST were excluded from analysis. Six sections of C7 spinal cord per animal were averaged to generate the axon sprouting index per animal.

**Behavioral analyses**. Behavioral tests were done with the observer blind to genotypes. Two motor tasks were used to assess motor function before and after cerebral cortical photothrombosis. To evaluate gross motor coordination, mice were placed on a five-lane elevated rotating rod (IITC Life Science), facing away from the observer while walking on the rod. Rotarod was set with starting speed of 4 rpm, constant acceleration of 0.2 rpm/s, and top speed of 44 rpm. Maximum test duration was 300 s. Mice were scored based on time to fall or holding onto the rod without moving. Three trials were averaged for a final score per mouse per session. Mice had pre-injury rotarod training once a day, consecutively for 5 days before injury. Post-injury testing was done at 1, 2, 4, 6, and 8 weeks after unilateral photothrombosis. Post-injury end time of each animal was normalized to its own pre-injury end time, with the pre-injury baseline performance represented as 1 on quantification. To evaluate skilled forelimb stepping, regular horizontal ladder was used. Mice were filmed (60 fps, interlaced, and rendered to 30 fps playback) walking across an 81 cm horizontal ladder with 1.5 cm regularly spaced rungs (Farr et al., 2006). Paw placement of each forelimb was video-scored and defined as follows: correct placement (score of 6); partial placement (score of 5); misses (scores of 0–4). Each animal had three trials per day of testing. Number of steps from each placement type was expressed as a percentage of total steps. Three trials were averaged per mouse per session. Mice had pre-injury training three days before injury. Post-injury testing was done at 1, 2, 4, 6, and 8 weeks after unilateral photothrombosis.

**Statistical analyses**. One-way or two-way ANOVA analysis was applied depending on the experimental design (see Figure Legends), using GraphPad Prism software. *Post-hoc* comparisons were performed only when a main effect showed statistical significance. Bonferroni correction was used to adjust *p* value of multiple comparisons. All graphs show mean with SEM. *N* represents the number of mice per genotype or treatment group. See figure legends for exact n and *p* values, and statistical test applied.

## Data availability statement

The original contributions presented in this study are included in the article. Further inquiries can be directed to the corresponding authors.

## Ethics statement

The animal study was reviewed and approved by Institutional Animal Care and Use Committee, University of Kentucky and UT Southwestern Medical Center.

## Author contributions

MC conceived the study. MC and MG organized the study, and wrote the manuscript. MC, LI, and EP planned and performed the experiments. MC, XK, RT, and NG performed sample and data collection. MC, NG, MR, and WF performed the data analyses. All authors contributed to manuscript reading and approved submitted manuscript.
